# Symmetric stimulation of Hsp90 catalyzed ATP hydrolysis through enhanced active site gate dynamics

**DOI:** 10.1016/j.jbc.2025.110262

**Published:** 2025-05-21

**Authors:** Breanna Magnan, Thomas Dumont, Suad Rashid, Paul LaPointe, Leo Spyracopoulos

**Affiliations:** 1 Department of Biochemistry, University of Alberta, Edmonton, Alberta, Canada; 2 Department of Cell Biology, University of Alberta, Edmonton, Alberta, Canada

**Keywords:** ATPase, heat shock protein 90, chaperone, ^19^F NMR, enzyme kinetics, protein dynamics, molecular chaperone, molecular dynamics

## Abstract

Heat shock protein 90 (Hsp90) is a vital molecular chaperone that is essential for activating a diverse array of regulatory proteins through an ATP-dependent clamping cycle. The Hsp90 clamping cycle is driven by large-amplitude conformational changes within the N-terminal ATPase domain, including the release of an autoinhibitory N-terminal **β**-strap followed by a less well-characterized ATP gate rearrangement involving N-terminal helix 1. Here, we employed a combination of ^19^F NMR spectroscopy, molecular dynamics simulations, and ATPase assays to examine the effects of targeted **β**-strap and helix 1 mutations. Our findings reveal that targeted disruption of helix 1 packing against the ATPase domain accelerates clamp closure, symmetrically enhancing ATP hydrolysis for both subunits of the Hsp90 dimer, whereas activation by the Aha1 (activator of Hsp90 activity 1) cochaperone is disrupted. Decreasing the energy barrier associated with helix 1 release is a key step in modulating the energy landscape that governs the dynamics of the Hsp90 clamping cycle.

Heat shock protein 90 (Hsp90) is an essential and universally conserved molecular chaperone that stabilizes and activates a wide range of regulatory proteins, including various kinases and steroid receptors (1, 2, 3). The action of Hsp90 is dependent on an ATP-driven substrate clamping cycle that is regulated by a network of post-translational modifications and cochaperone proteins (4). Hsp90 is an obligate homodimer with each subunit composed of three independent domains including an N-terminal ATPase domain linked to a middle substrate-binding domain through an acidic, 60 residue linker, and a C-terminal dimerization domain (5). In the absence of nucleotide, Hsp90 adopts an open, C-terminally dimerized conformation that is competent to bind substrate proteins (6, 7). ATP binding induces a series of large-amplitude conformational changes that promote N-terminal dimerization as well as intersubunit and intrasubunit N–M association, which allow the dimer to adopt a compact, and catalytically active, clamp-closed state (1, 6, 7, 8, 9, 10). Compaction of the chaperone is relieved by ATP hydrolysis, which releases ADP and allows the chaperone to return to an open state (11). Absent cochaperones, or post-translational modifications, the “bind and release” cycle of Hsp90 is characterized by slow kinetics, with several steps requiring an energy expenditure of ∼20 kcal mol^−1^ (12, 13, 14). Notably, many of these conformational changes occur within the ATPase domain. Specifically, release of the N-terminal β-strap (residues 1–8) from the central β-sheet repositions helix 1 (residues 9–22) to the interface between N-terminal domains and drives closure of the ATP gate (residues 94–125). These rearrangements promote N-terminal dimerization through hydrophobic interactions between helix 1 of one subunit and helix 1 of the opposing subunit (10).

We recently developed a kinetic model for ATPase domain remodeling, identifying two important high-barrier conformational changes (15): the first step is characterized by N-terminal β-strap release, and a second less well-characterized conformational rearrangement involves gate release. The release of the N-terminal β-strap from the bulk of the ATPase domain in conjunction with the second conformational change facilitates ATP gate closure, alleviates autoinhibition of ATPase activity, and initiates the clamping cycle (15). The importance of the β-strap in the clamping cycle is further highlighted through studies which show the strap mediates interactions between the ATPase domain and the activating cochaperone Aha1 (activator of Hsp90 activity 1) (15). Consistent with these findings, complete strap removal leads to increased ATPase activity (16) with reduced Aha1 stimulation (17), whereas strap destabilization through F6D/F8D double mutations abolishes both ATPase activity and Aha1 stimulation (15). Interestingly, we found that in the context of intact, heterodimeric WT:F6D/F8D Hsp90, the F6D/F8D subunit asymmetrically stimulates the ATPase activity of a WT subunit (15). Whilst the molecular mechanism of asymmetric stimulation through strap destabilization is somewhat unclear, our previous results for heterodimeric WT:F6D/F8D Hsp90 suggest it is driven by intersubunit N–M interactions between helix 1 of the F6D/F8D subunit and the closed ATP gate of the WT subunit (15).

Our goal in this study was to develop mechanistic insights into the aforementioned second high-energy conformational rearrangement within the ATPase domain and to better understand the associated role in gate release. Our previous ^19^F NMR and molecular dynamics (MD) studies suggested that ATP gate closure, that is, release of the gate from the ATPase domain core, may be facilitated by the disruption of interactions between helix 1 from the ATPase domain core and helix 5 from the ATP gate (15). Additional studies involving mutations within, or the removal of helix 1, have also implied a key role in modulating Hsp90 ATPase activity. More specifically, T22E, T22I, or T22F mutations increase ATPase activity (18, 19, 20), whereas removal of the first 16 (Δ16) or 24 (Δ24) N-terminal residues abolished activity (16, 21). In addition, the T22E mutation reduces the ability of Hsp90 to be stimulated by Aha1 (20), implicating helix 1 in mediating cochaperone interactions.

To explore the role of helix 1 and its coupling to the β-strap in the mechanism of gate release and clamp cycle activation, we focused our studies on L18, a key helix 1 residue that participates in hydrophobic interactions with helix 5 of the ATP gate to stabilize the open gate conformation within an individual subunit. In addition, within intact, dimeric Hsp90, ATP gate closing results in intrasubunit interactions shifting to intersubunit interactions with L18 within helix 1 from one subunit participating in cross-subunit helix 1–helix 1 hydrophobic interactions, which stabilize the catalytically active compact state of the intact chaperone (10, 22). We used site-directed mutagenesis to generate an N-domain L18D mutant that disrupts these hydrophobic intrasubunit and intersubunit hydrophobic interactions. We also generated an N-domain strap/helix 1 F6D/F8D/L18D mutant to probe the coupling between these ATPase domain regions. We employed ^19^F NMR spectroscopy and MD simulations to gain insights into the structural and dynamic impacts associated with helix 1 and strap/helix mutants. To probe clamp closure and ATP hydrolysis in the intact chaperone, we blended ^19^F NMR spectroscopic studies and ATPase assays for intact L18D and F6D/F8D/L18D Hsp90 mutant homodimers and mutant/WT heterodimers. Collectively, our results provide a mechanistic framework to better understand how the high-barrier conformational changes within the ATPase domain drive the Hsp90 clamping cycle and have important implications for the biological function and internal regulation of Hsp90.

## Results

### Destabilization of helix 1–helix 5 interactions within the ATPase domain alters conformational cycling

We previously identified two high-barrier conformational changes that facilitate gate closure within the ATPase domain of Hsp90 (Hsp90N) (15). Specifically, ATP binding promotes release of the N-terminal β-strap as a first step, with a subsequent conformational rearrangement that facilitates ATP gate closure. The molecular basis of the second step is not clearly understood, though we speculate that release of helix 1 from the ATPase core represents a key contribution, given structural observations that show that upon disruption of intrasubunit helix 1–helix 5 interactions, the ATP gate is released, and closes over the active site, with helix 1 concomitantly participating in intersubunit helix 1–helix 1 interactions that stabilize the compact, catalytically active conformation of the intact chaperone (Fig. S1, *A* and *B*) (10, 22).

**Figure 1:**
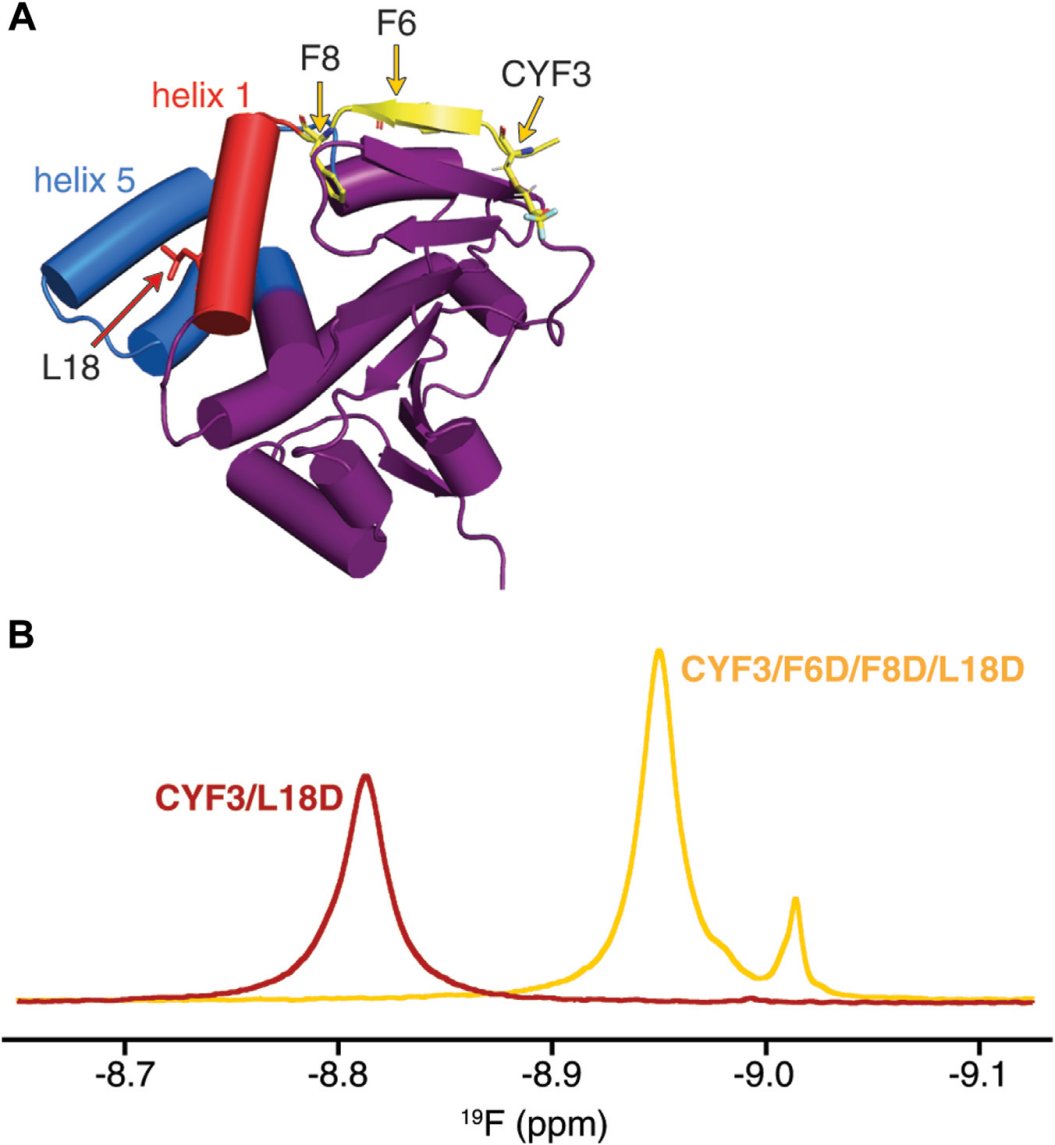
**^19^F chemical shift changes resulting from N-terminal β-strap and α-helix 1 mutations.***A*, a structural model for Hsp90N-CYF3 in the apo state (Protein Data Bank ID: 1AH6) with the main chain in the schematic representation. The N-terminal β-strap is shown in *yellow*, helix 1 is shown in *red*, and the ATP gate is shown in *blue*. CYF3, F6, F8, and L18 residues are shown in the *stick representation*. *B*, ^19^F NMR spectra for Hsp90N-CYF3/L18D (*red*) and Hsp90N-CYF3/F6D/F8D/L18D (*yellow*) at 25 °C in the absence of nucleotide. A superimposition of ^19^F NMR spectra for Hsp90N-CYF3, -CYF3/L18D, -CYF3/F6D/F8D, -CYF3/F6D/F8D/G123P, and -CYF3/F6D/F8D/L18D is shown in Figure S1. Hsp90, heat shock protein 90.

To test whether disruption of the helix 1–helix 5 hydrophobic interaction is important for the shift to the compact, catalytically active state, we introduced an L18D mutation into Hsp90N-CYF3 (Fig. 1*A*). For this mutant, we observed a ^19^F NMR peak (Fig. 1*B*) that displays moderate changes in the ^19^F chemical shift for CYF3 within the N-terminal β-strap, in comparison to our previous results for Hsp90N-CYF3 (Fig. S1*C*) (15). In addition, the *T*_2_ value of 33.3 ± 0.3 ms for CYF3/L18D is similar to the value of 32.2 ± 0.3 ms for CYF3 (15), suggesting that the β-strap is stably attached to the bulk of the ATPase domain for L18D. In agreement, MD simulations for CYF3 and CYF3/L18D N-domain mutants exhibit stable N-terminal β-straps (Fig. S2, *A* and *B*). However, the simulations also show that L18D increases the main chain flexibility for both helix 1 and the ATP gate (Fig. S2, *A* and *B*). Notably, we observed a three-fold reduction in positively correlated motions between helix 1 and the ATP gate with the values for total correlation decreasing from +21.6 for CYF3 to +6.7 for CYF3/L18D in the absence of nucleotide. These motions become further anticorrelated upon binding ATP with the total correlation decreasing from +1.6 for WT to −17.2 for the L18D mutant.

We next examined structural and dynamic coupling between the N-terminal β-strap and helix 1 by introducing destabilizing F6D/F8D strap mutations into Hsp90N-CYF3/L18D. In our previous studies, F6D/F8D double mutations increased the flexibility of the N-terminal β-strap and the ATP gate by reducing the energy required for β-strap release, allowing for more rapid cycling between the gate open and gate closed conformations (15). Notably, the ^19^F NMR spectra for CYF3/F6D/F8D (15) at 25 °C and CYF3/F6D/F8D/L18D at 10 °C (Figs. S1*C* and S3) contain multiple NMR resonances with comparable ^19^F chemical shifts. We putatively assigned these resonances to distinct conformations within the ATPase domain based on our previous analyses of temperature-dependent ^19^F NMR lineshapes and Carr–Purcell–Meiboom–Gill (CPMG) relaxation dispersion data for Hsp90N-CYF3, CYF3/F6D/F8D, and CYF3/F6D/F8D/G123P mutants (Figs. S1*C* and S3) (15). At 25 °C, we can observe two distinct conformations within the ^19^F NMR spectrum of Hsp90N-CYF3/F6D/F8D/L18D: a predominant and more populated lower energy ground state (G-state or strap-on state) and a second less populated, higher energy strap-off state (S-state) (Fig. 1*B*). The *T*_2_ value of 64.1 ± 0.4 ms for the G-state of CYF3/F6D/F8D/L18D compared with the value of 45.8 ± 0.6 ms for the G-state of CYF3/F6D/F8D (15) is indicative of an increase in N-terminal β-strap flexibility for CYF3/F6D/F8D/L18D. The less intense ^19^F NMR peak for the S-state of CYF3/F6D/F8D/L18D (Fig. 1*B*) displays a longer *T*_2_ value of 125 ± 1 ms, similar to the value of 110 ± 2 ms we previously measured for the S-state of the CYF3/F6D/F8D/G123P mutant (15). For this mutant, our combined MD and ^19^F NMR results from previous studies suggest that when the pre–proline V122 main chain ω dihedral angle adopts the *cis* conformation, the N-terminal β-strap is released from the domain, as G123 forms part of the C-terminal hinge of the ATP gate (Fig. S1*C*) (15). Consistent with a strap-off state evident within ^19^F NMR spectra of CYF3/F6D/F8D/L18D, MD simulations for CYF3/F6D/F8D and CYF3/F6D/F8D/L18D N-domain mutants exhibit a highly flexible and unstructured β-strap that is predominantly dissociated from the ATPase core (Fig. S4, *A* and *B*). However, MD simulations also reveal that L18D fluctuates between strap-on and strap-off states within the F6D/F8D N-domain mutant (Fig. S4*B*). Furthermore, we observe distinct correlated motions between helix 1 and the ATP gate with highly positively correlated motions for apo CYF3/F6D/F8D (total correlation of +58.3), which are highly anticorrelated in CYF3/F6D/F8D/L18D (total correlation of −104.9). For ATP binding, the total correlation is reduced from +58.3 to +33.9 for CYF3/F6D/F8D, whereas for CYF3/F6D/F8D/L18D, the total correlation decreases from −104.9 to −43.2.

**Figure 2:**
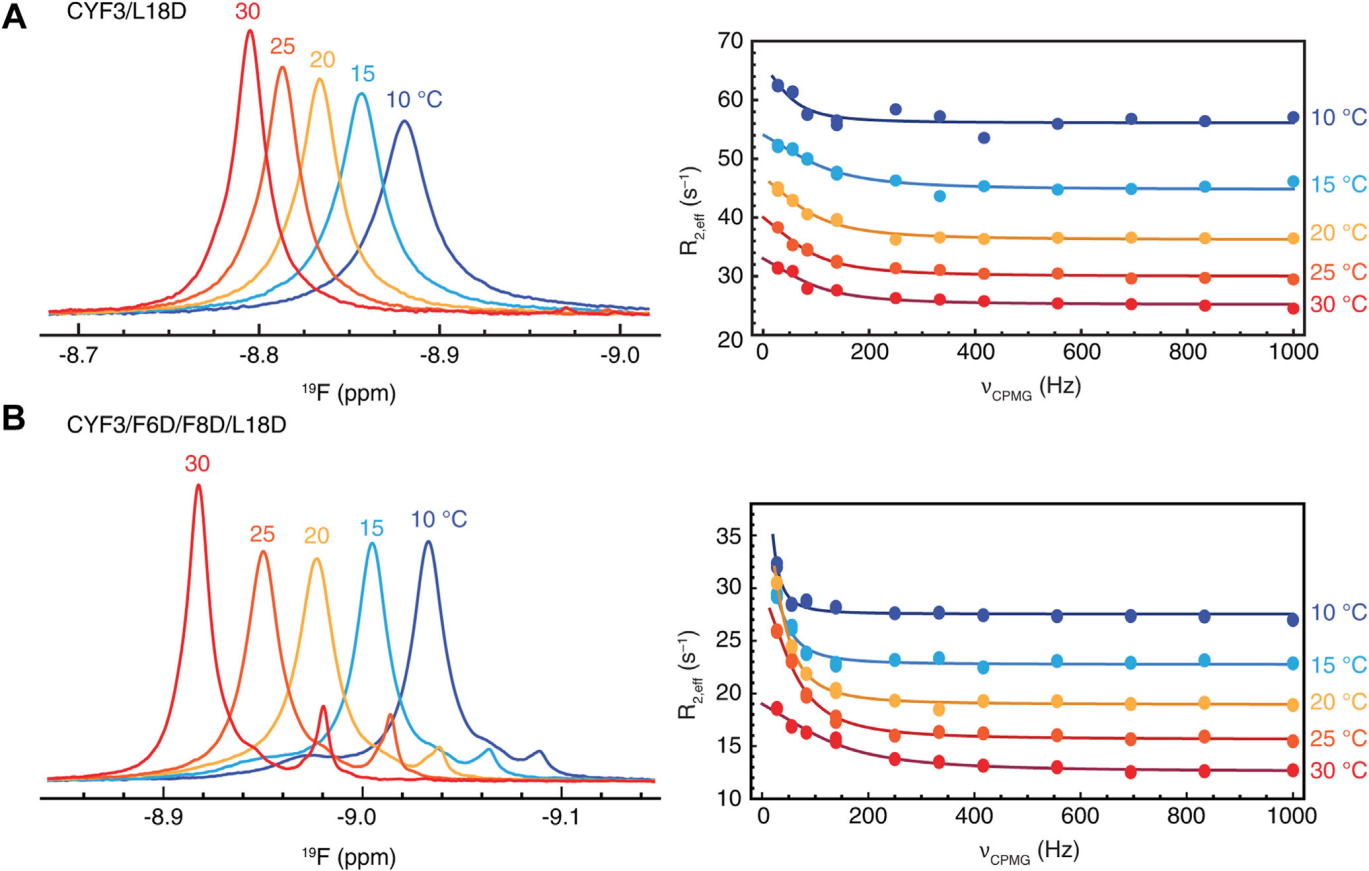
**Temperature-dependent ^19^F NMR resonances and CPMG relaxation dispersion profiles reveal conformational exchange processes for the N-terminal β-strap and helix 1.**^19^F NMR spectra (*left panel*) and CPMG relaxation dispersion profiles (*right panel*) at 657 MHz for Hsp90N-CYF3/L18D (*A*) and Hsp90N-CYF3/F6D/F8D/L18D (*B*) recorded at various temperatures in the absence of nucleotide. For the most intense resonance, temperature-dependent NMR relaxation dispersion data (*solid dots*) were fit (*solid lines*) to a two-state model for fast exchange. CPMG, Carr–Purcell–Meiboom–Gill; Hsp90, heat shock protein 90.

To characterize the kinetics of N-terminal β-strap release for Hsp90N-CYF3/L18D and CYF3/F6D/F8D/L18D, we analyzed ^19^F CPMG NMR relaxation dispersion data at multiple temperatures using a two-state model with fast exchange kinetics. The CPMG profiles of Hsp90N-CYF3/L18D (Fig. 2*A*, Table S1) reveal a fast exchange process that was not previously observed for Hsp90N-CYF3 (15). In contrast, ^19^F CPMG relaxation dispersion experiments for Hsp90N-CYF3/F6D/F8D/L18D display exchange profiles consistent with the introduction of slower conformational processes, or alternatively, the slowing of these processes compared with those observed for the L18D mutant (Fig. 2*B*, Table S1).

### ^19^F NMR spectra for intact Hsp90 L18D mutants reveal the nature of intersubunit interactions

In addition to our ATPase domain studies, we introduced L18D and F6D/F8D/L18D mutations into Hsp90-CYF61 to characterize the role of helix 1 in the context of intact Hsp90. We previously identified three conformational states for intact Hsp90-CYF61 using ^19^F NMR spectroscopy (15): intrasubunit association of the N and M domains (NM-state), intrasubunit dissociation of the N and M domains (free N-domain), and a clamp-closed state, where the N, M, and C domains are closely associated with their respective domain in the partner subunit of the dimer (Fig. S5*A*). Furthermore, we previously showed that ^19^F NMR spectra and dynamic NMR studies for intact Hsp90-CYF61 report on the conformational shift to the compact state upon binding the nonhydrolyzable ATP analog adenylyl-imidodiphosphate (AMP-PNP) (Fig. S5*B*) (14). In this study, we generated an Hsp90-CYF110 mutant, for which the ^19^F NMR reporter probe is situated at the apex of the ATP gate (Fig. S5, *A* and *C*). Chemical shifts observed within ^19^F NMR spectra for Hsp90-CYF110 indicate that the gate adopts a different conformation upon binding of AMP-PNP, and becomes more flexible, as the NMR resonance narrows (Fig. S5*C*). This interpretation of increased flexibility, at least on the nanosecond to picosecond timescale, upon AMP-PNP binding is commensurate with flat CPMG dispersion profiles for both the apo and bound states (Fig. S5*D*). In addition, ^19^F dynamic NMR spectra for Hsp90-CYF110 upon binding AMP-PNP show similar interconversion rates between the NM and clamp-closed states as Hsp90-CYF61 (Fig. S5*E*) (14). The combined results of our previous ^19^F NMR studies of Hsp90-CYF61 and -CYF110 in this study indicate that both probes report on the same conformational process; a slow shift to a gate closed state that accompanies adoption of the closed, compact, and catalytically active state upon nucleotide binding.

**Figure 3:**
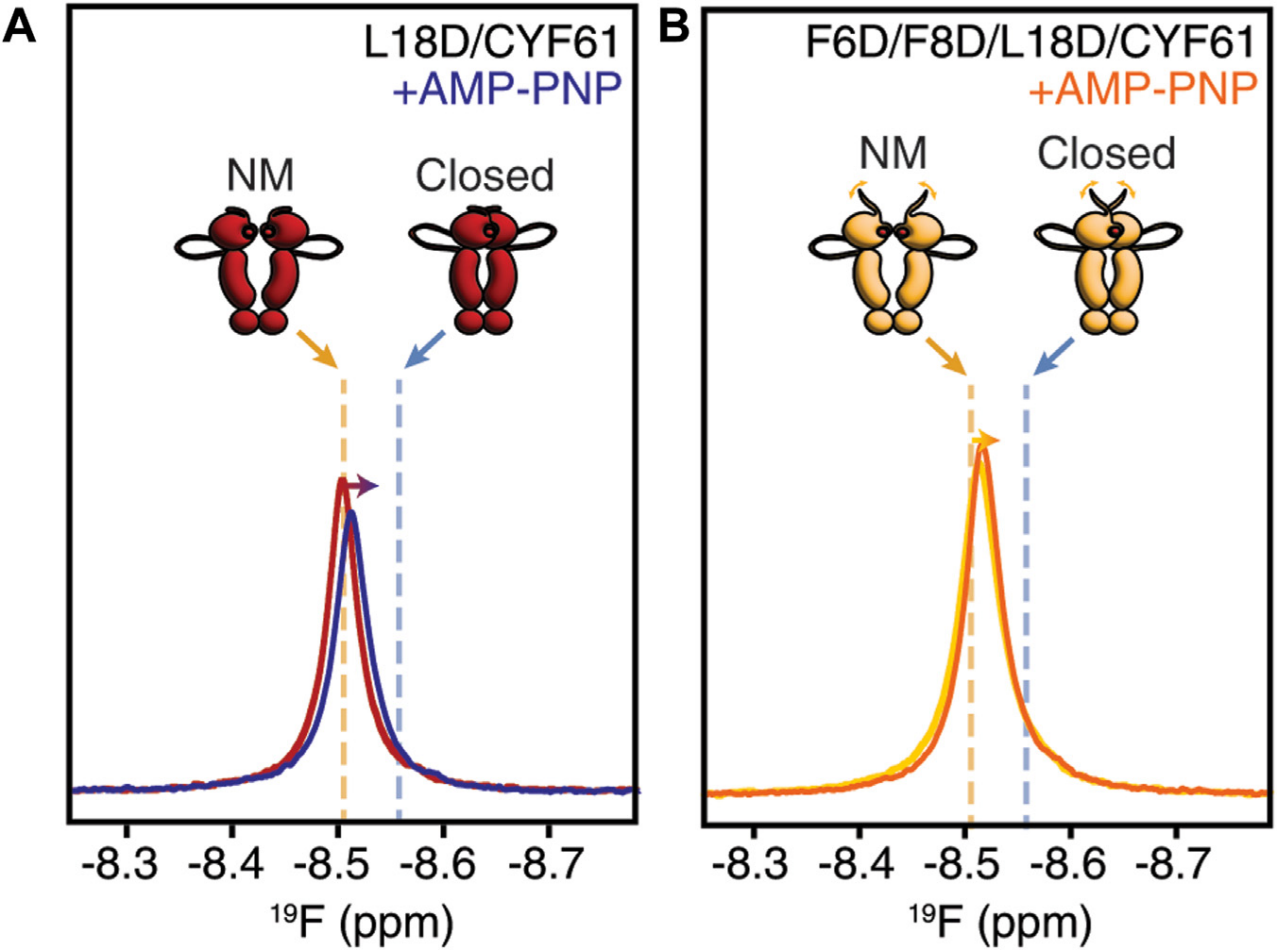
**^19^F NMR spectra for intact Hsp90 L18D and F6D/F8D/L18D mutants reveal the nature of intersubunit interactions.***A*, ^19^F NMR spectra for Hsp90-L18D/CYF61 (*red*) and (*B*) Hsp90-F6D/F8D/L18D/CYF61 (*yellow*) in the absence and presence of AMP-PNP. ^19^F chemical shifts for the NM-associated and clamp-closed states for Hsp90-CYF61 are shown as *dashed lines*. AMP-PNP, adenylyl-imidodiphosphate; Hsp90, heat shock protein 90.

For apo Hsp90-L18D/CYF61, we observed a single ^19^F NMR peak that corresponds to a weighted average between the NM-associated and free N-domain states (Fig. 3*A*). The addition of AMP-PNP induces moderate spectral changes, specifically, an upfield shift that likely represents a weighted average between the NM and clamp-closed states, as the build-up of clamp-closed state is too rapid to be observed in ^19^F dynamic NMR experiments, unlike the slower build-up of clamp-closed state observed for Hsp90-CYF61 (14). Assuming fast exchange between NM and clamp-closed states, the weighted chemical shift for Hsp90-L18D/CYF61 upon AMP-PNP binding indicates that the clamp-closed state is populated to ∼15%, compared with a value of ∼60% for Hsp90-CYF61. Similarly, the ^19^F NMR spectrum of Hsp90-F6D/F8D/L18D/CYF61 displays a single NMR peak that is a weighted average between the NM and clamp-closed states; however, addition of AMP-PNP does not induce appreciable spectral changes (Fig. 3*B*). These results are indicative of fast exchange between the NM and clamp-closed states for both L18D and F6D/F8D/L18D.

### L18D homodimers have enhanced ATPase activity compared with WT

**Figure 4:**
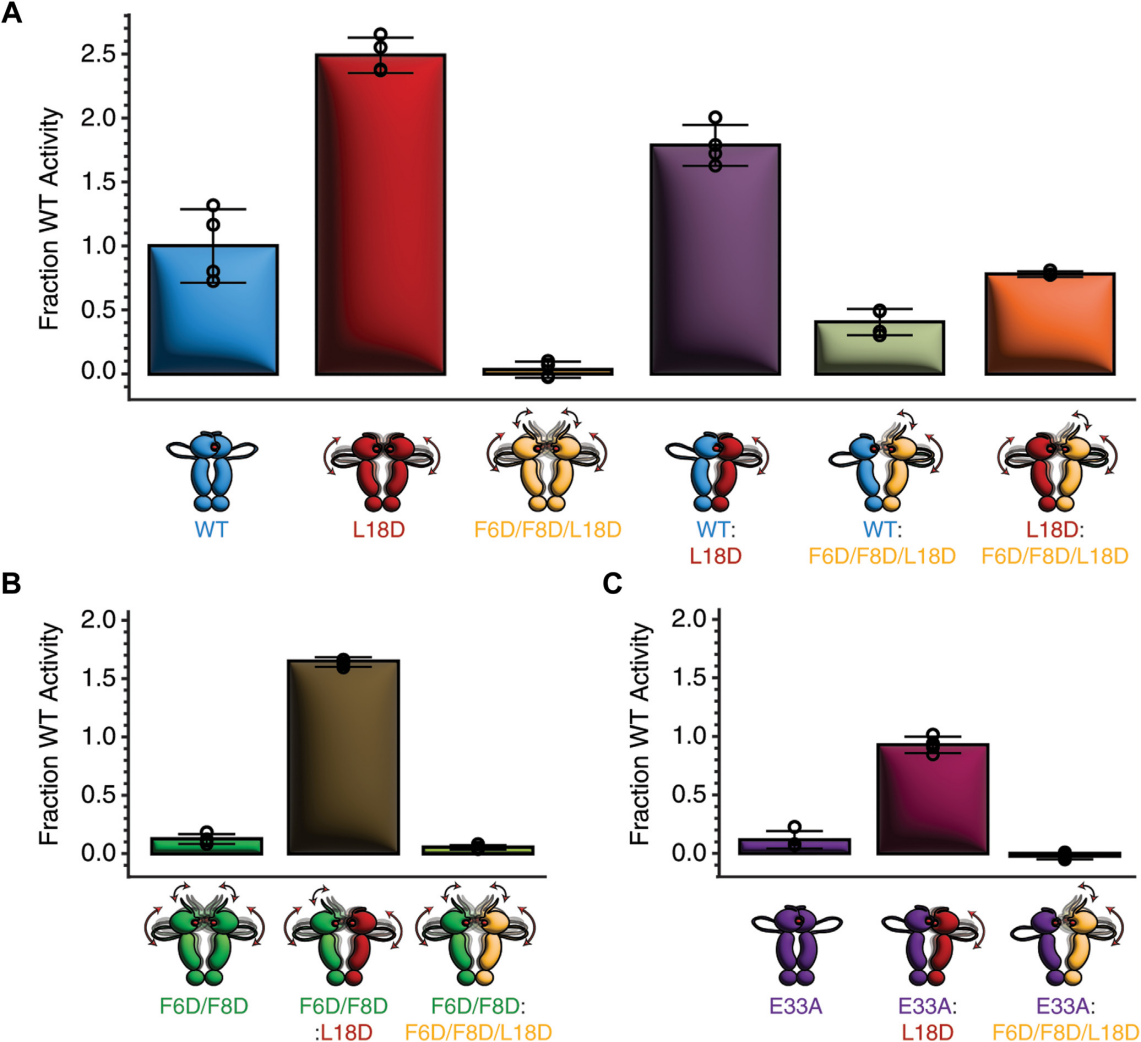
**L18D homodimers and heterodimers accelerate Hsp90 ATPase activity compared with WT.***A*, ATPase activity for WT (*blue*), L18D (*red*), and F6D/F8D/L18D (*yellow*) Hsp90 homodimers and heterodimers. *B*, ATPase activity for F6D/F8D:L18D (*green:red*) and F6D/F8D:F6D/F8D/L18D (*green:yellow*) Hsp90 heterodimers. *C*, ATPase activity for E33A:L18D (*purple:red*) and E33A:F6D/F8D/L18D (*purple:yellow*) Hsp90 heterodimers. The ATPase activity is shown as a fraction of WT homodimer activity with error bars representing the standard deviation for four repetitions. Hsp90, heat shock protein 90.

We further assessed the role of helix 1 in Hsp90-catalyzed ATP hydrolysis using an ATP-regenerating, coupled enzyme assay (23). Hsp90-L18D homodimers demonstrate a ∼2.5-fold increase in ATPase activity compared with WT homodimers, whereas F6D/F8D/L18D homodimers lack ATPase activity (Fig. 4*A*). We also examined cross-subunit interactions for intact Hsp90 by examining ATPase activities for heterodimers consisting of 1:1 stoichiometric mixtures of WT:L18D or WT:F6D/F8D/L18D subunits. The measured ATPase activities for the various heterodimers are roughly the average value of the WT homodimer and the respective L18D or F6D/F8D/L18D homodimer activities (Fig. 4*A*). This finding suggests that cross-subunit interactions do not contribute to either stimulation or repression of ATP hydrolysis beyond the measured synergistic effects expected for WT dimers (5). Interestingly, these average ATPase activities are not observed within L18D:F6D/F8D/L18D heterodimers, rather we observe a value smaller than the average, and which is a 20% reduction compared to WT ATPase activity, which suggests that cross-subunit helix 1–helix 1 interactions are important for hydrolysis, and are likely diminished for L18D:F6D/F8D/L18D heterodimers as a combined effect as the result of β-strap destabilization for one subunit because of the F6D/F8D double mutation, and helix 1–helix 1 interactions destabilized by L18D in both subunits (Fig. 4*A*). Furthermore, while we previously observed that F6D/F8D double mutations induce ATPase activation for the WT protomer within asymmetric WT:F6D/F8D heterodimers (15), this was not observed for WT:F6D/F8D/L18D heterodimers, suggesting that helix 1 is important for cross-subunit activation and stabilization (Fig. 4*A*). Thus, we assessed whether an L18D subunit can be asymmetrically activated by an F6D/F8D subunit. We find an ∼1.6-fold increase in ATPase activity for F6D/F8D:L18D heterodimers compared with WT homodimers (Fig. 4*B*). This increase is similar to that previously measured for WT:F6D/F8D heterodimers (15). However, the activity of F6D/F8D:L18D heterodimers is not stimulated above L18D homodimer activity, with a ∼1.4-fold increase in L18D subunit activity for the F6D/F8D:L18D heterodimer compared with a ∼3-fold increase in WT subunit activity for the WT:F6D/F8D heterodimer. We did not observe ATPase stimulation for F6D/F8D:F6D/F8D/L18D heterodimers (Fig. 4*B*).

In order to confirm which subunit within Hsp90 heterodimers is responsible for ATPase activity, we generated heterodimers containing a catalytically inactive E33A subunit (24). Within E33A:L18D heterodimers, the L18D subunit shows ATPase activity similar to that for WT homodimers (Fig. 4*C*), whereas assays employing E33A:F6D/F8D/L18D heterodimers do not show appreciable ATPase activity (Fig. 4*C*).

### Quantification of ATPase activity modulation by symmetric and asymmetric subunit composition

**Figure 5:**
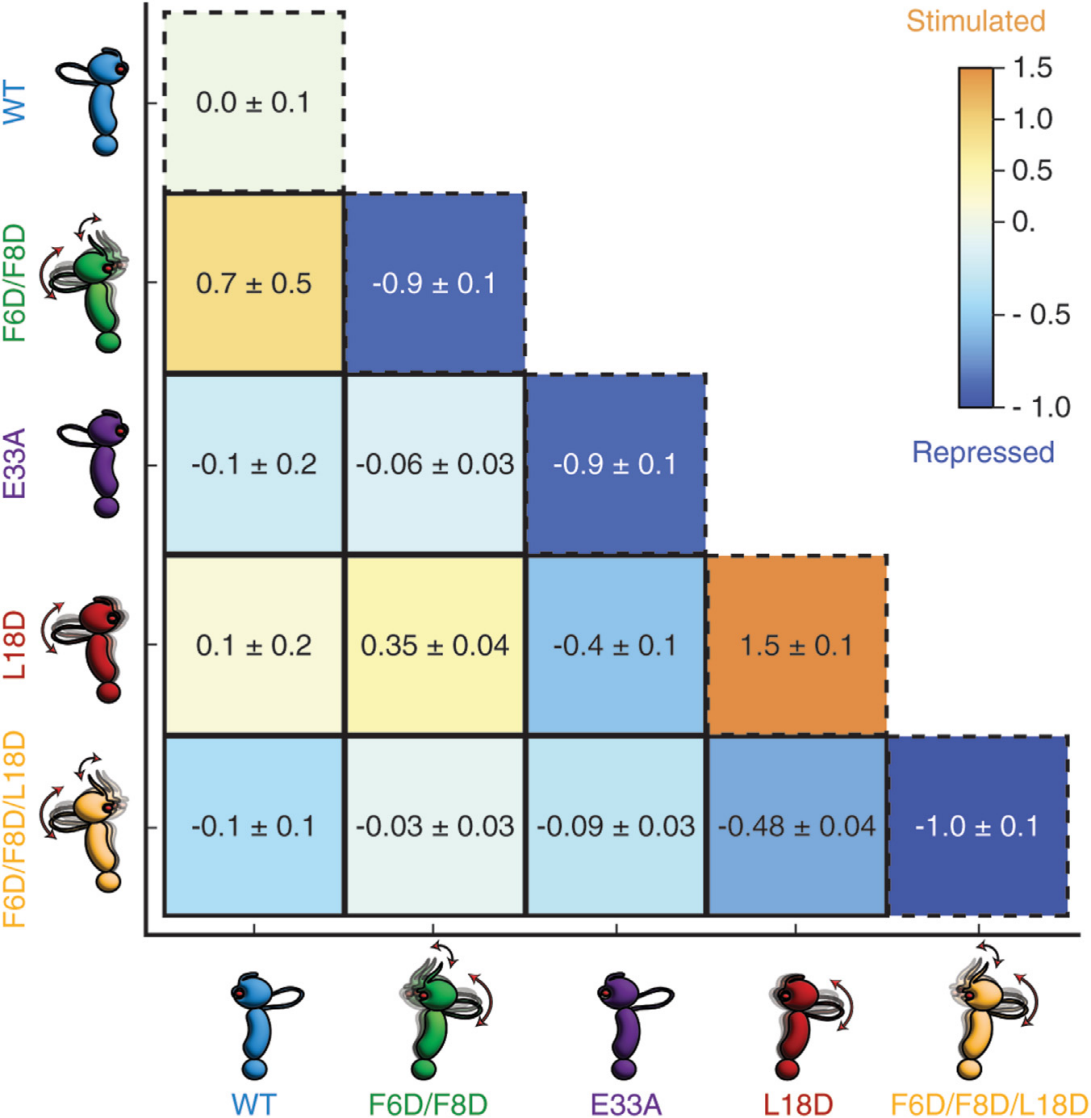
**Modulation of Hsp90 ATPase activity by symmetric and asymmetric subunit composition.** Lower triangular matrix for changes in fractional WT ATPase activity for various pairs of Hsp90 subunits. Changes in activity for symmetric subunits (Δ_symm_) for Hsp90 homodimers are given by the diagonal elements (*dashed boxes*), whereas changes in activity for asymmetric subunits (Δ_asymm_) for Hsp90 heterodimers are given by the off-diagonal elements (*solid boxes*). Values of Δ_symm_ and Δ_asymm_ that represent stimulated ATPase activity relative to WT are shown in *orange*, values for repressed ATPase activity are shown in *blue*, and activity at the WT level is shown in *gray*. Hsp90, heat shock protein 90.

To view our ATPase activity results in a clearer context, we developed two parameters, Δ_symm_ and Δ_asymm_ (see the *Experimental procedures* section), which provide a means to analyze the fractional WT ATPase activity of homodimers resulting from a single subunit mixture, as well as the purely heterodimer component from a 1:2:1 mixture of homodimer1:heterodimer:homodimer2 obtained from 1:1 stoichiometric mixing of two different subunits (Fig. 5). To interpret these parameters, we expect WT homodimers have Δ_symm_ = 0 by definition, whereas if the fractional WT ATPase activities are repressed, Δ_asymm_ and Δ_symm_ are negative with values <0 down to a minimum of −1. For stimulation of fractional WT ATPase activity, the values for Δ_asymm_ and Δ_symm_ are positive, with values >0, up to the observed increase in fractional WT ATPase activity. As evident in Figure 5, WT homodimers have Δ_symm_ = 0, whereas F6D/F8D, F6D/F8D/L18D, and E33A homodimers are largely fully repressed with Δ_symm_ ∼ −1, and with L18D homodimers showing the largest stimulation with Δ_symm_ = +1.5. Asymmetric stimulation of either a WT subunit or an L18D subunit by a catalytically defunct F6D/F8D subunit is evident through Δ_asymm_ values of +0.7 and +0.35, respectively (Fig. 5). All other heterodimers show either no significant differences from fractional WT ATPase activity or a repression of activity with Δ_asymm_ values <0.

### Aha1 stimulation of L18D heterodimers provides insight for interactions between Aha1 and the ATPase domain of Hsp90

**Figure 6:**
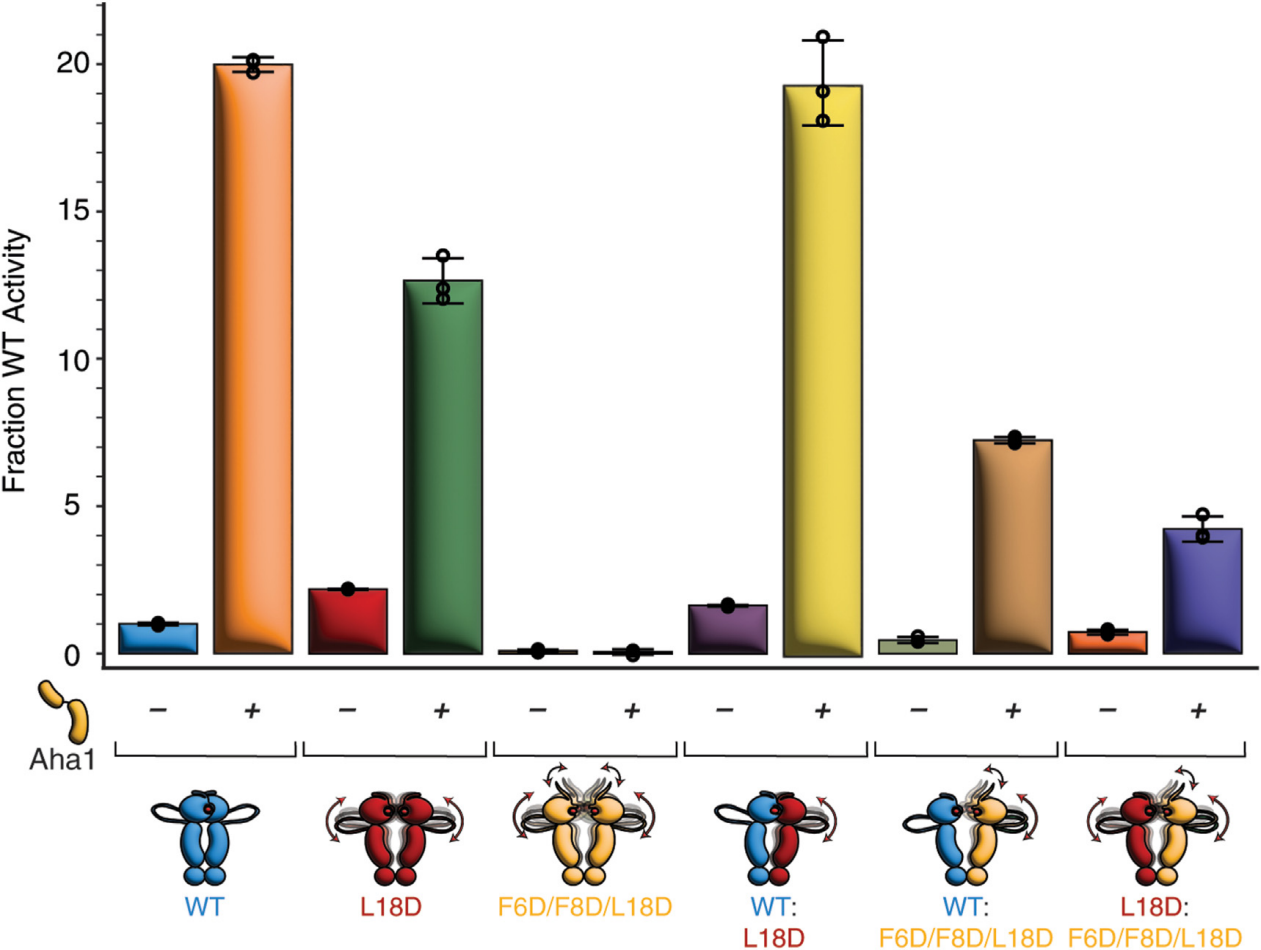
**Maximal stimulation by Aha1 requires a WT subunit.** ATPase activity for WT, L18D, and F6D/F8D/L18D homodimers and heterodimers in the absence and presence of Aha1. The ATPase activity is shown as a fraction of WT homodimer activity with error bars representing the standard deviation for three repetitions. Aha1, activator of Hsp90 activity 1.

We explored the ability of Aha1 to stimulate ATPase activity in L18D mutants using ATP-regenerating, coupled enzyme assays (23). For WT homodimers, we found that Aha1 induces an ∼20-fold stimulation in ATPase activity (Fig. 6, Table S2), consistent with previous studies (25, 26, 27), whereas stimulation is reduced to approximately six-fold for L18D homodimers and absent for F6D/F8D/L18D homodimers (Fig. 6, Table S2). Furthermore, heterodimers possessing a WT subunit have higher levels of Aha1 stimulation, with ∼12- and ∼16-fold stimulation for WT:L18D and WT:F6D/F8D/L18D heterodimers, respectively (Fig. 6, Table S2). In contrast, ATPase stimulation is reduced to approximately six-fold for L18D:F6D/F8D/L18D heterodimers (Fig. 6, Table S2). Interestingly, Aha1-stimulated ATPase activity for WT:L18D heterodimers is comparable to Aha1 stimulation for WT. These results agree with our previous assessment that one subunit must possess a stable β-strap for effective Aha1 stimulation (15), with the reduction in Aha1 stimulation for L18D homodimers indicating that helix 1 has a role in mediating stimulation.

## Discussion

**Figure 7:**
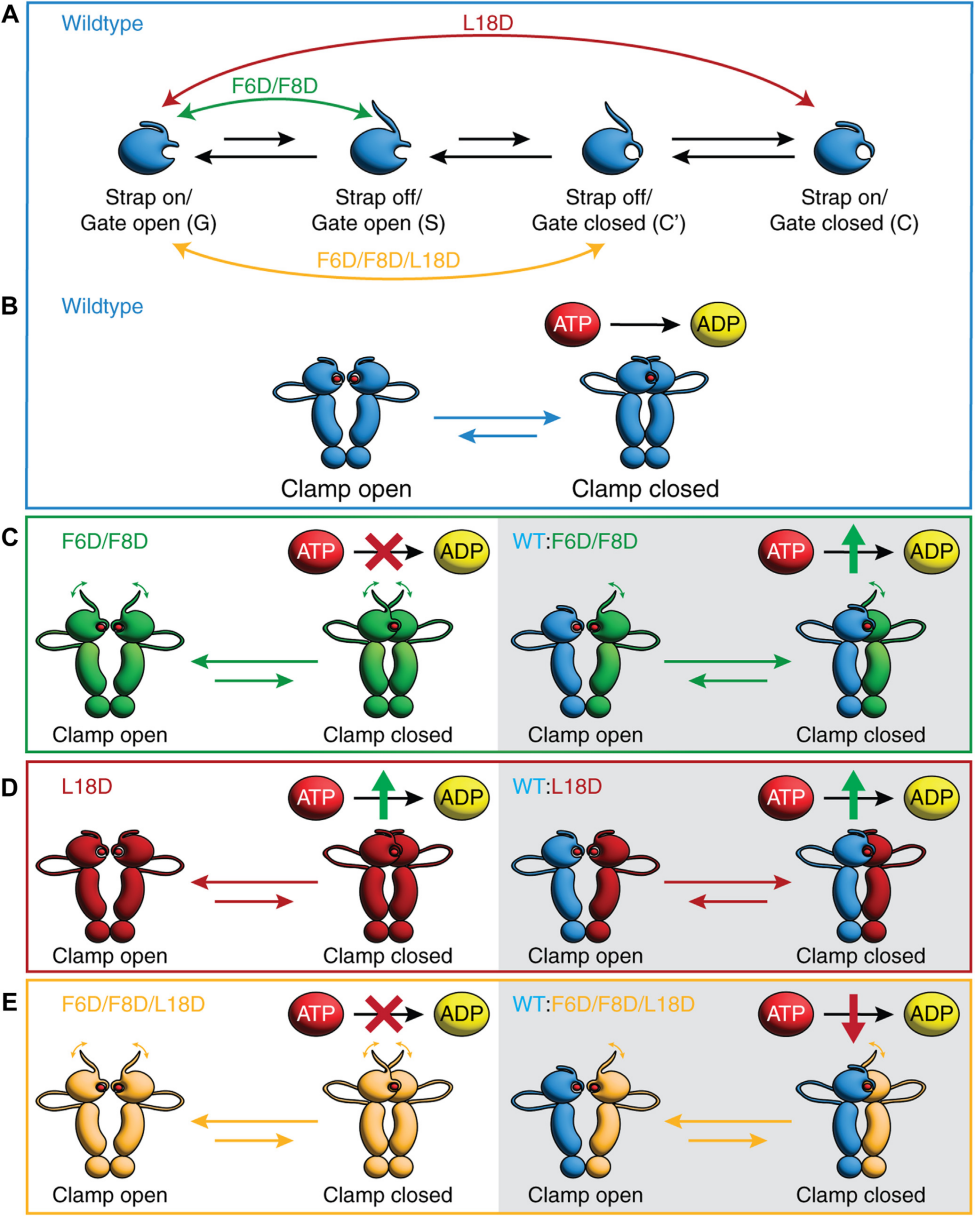
**N-terminal β-strap and helix 1 influence the Hsp90 clamping cycle.***A*, schematic diagram depicting conformational cycling within the ATPase domain of WT Hsp90. Conformational rearrangements that are accelerated by mutation are indicated by *green* (F6D/F8D), *red* (L18D), or *yellow* (F6D/F8D/L18D) *double-headed arrows*. *B*, schematic diagram of intact WT Hsp90 cycling between the clamp-open and -closed conformations. *C*–*E*, schematic diagrams depicting the impact of F6D/F8D (*C*, *green*), L18D (*D*, *red*), and F6D/F8D/L18D (*E*, *yellow*) mutations on conformational cycling of intact Hsp90 within homodimers (*left*) and WT:mutant heterodimers (*right*). Effects of mutations relative to WT homodimer ATP hydrolysis are shown as a *red cross* (no activity), *green up arrow* (increased activity), or *red down arrow* (decreased activity). Hsp90, heat shock protein 90.

Hsp90 functions to chaperone the folding and stability of numerous substrate proteins involved in a variety of cellular processes (1, 2). The ability of Hsp90 to process substrate proteins is dependent on an ATP-driven clamping cycle (11). In the absence of nucleotide, Hsp90 adopts a flexible, open conformation that is free to bind substrate. ATP binding induces a series of high-barrier conformational rearrangements within the N-domain of Hsp90 (Fig. 7*A*). These rearrangements promote intersubunit N–N interactions that enable the chaperone to adopt a stable, compact, and catalytically active, clamp-closed conformation, which binds and partly encloses substrates (Fig. 7*B*). The high-barrier conformational rearrangements within the ATPase domain are rate limiting in the Hsp90 substrate clamping cycle and similar in magnitude to the stability of globular proteins (28, 29, 30). The implication, therefore, is that cochaperone interactions or post-translational modifications of Hsp90 are likely to be essential modulators that reduce the energetic cost of ATPase strap release and gate closure, such that Hsp90 can effectively chaperone substrate proteins.

In our previous studies (15), we demonstrated that destabilization of the ATPase β-strap through F6D/F8D double mutations reduces the energy required for strap release (Fig. 7*A*), a key conformational rearrangement that relieves autoinhibition, and initiates the chaperone clamping cycle. In this study, we explored the molecular basis of a second high-energy conformational change within the ATPase domain by specifically disrupting helix 1-mediated stabilization of the open conformation of the ATP gate through an L18D mutation. The helix 1 L18D mutation results in increased ATP gate dynamics, with the β-strap remaining closely associated with the central β-sheet of the ATPase domain (Fig. 7*A*). In addition, combining L18D with F6D/F8D into a triple mutant induces changes in both the dynamics of the strap and ATP gate (Fig. 7*A*), as evidenced through our NMR studies of F6D/F8D/L18D, which reveal multiple ATPase domain conformations, including strap-off states that are otherwise hidden in WT spectra. These findings are consistent with a reduction in energy required for the ATPase domain to adopt a strap-off, gate-closed conformation for the L18D and F6D/F8D/L18D mutants.

For Hsp90 homodimers, the acceleration of β-strap dynamics for F6D/F8D double mutants (Fig. 7*C*) (15), enhanced ATP gate dynamics for L18D (Fig. 7*D*), or enhanced β-strap and ATP gate dynamics for F6D/F8D/L18D (Fig. 7*E*), are all associated with an acceleration in global conformational changes for intact Hsp90, with our NMR studies indicating rapid exchange between the clamp-open and clamp-closed states. The alterations in ATPase domain dynamics for these mutant subunits have different implications for ATP hydrolysis. We have previously shown that while F6D/F8D homodimers lack ATPase activity, enhancement of β-strap dynamics within the F6D/F8D subunit of a heterodimer stimulates ATP hydrolysis in the functional WT subunit (15) (Fig. 7*C*). In this study, we do not observe ATPase activity for F6D/F8D/L18D homodimers (Fig. 7*E*), whereas L18D homodimers have enhanced ATP hydrolysis compared with WT homodimers (Fig. 7*D*). Previous crystallographic studies have indicated that intrasubunit helix 1–helix 5 interactions are disrupted on ATP binding, concomitant with ATP gate closing and stabilization of the catalytically active state through intersubunit helix 1–helix 1 interactions. Furthermore, our ^19^F studies of AMP-PNP binding for Hsp90-CYF110 are consistent with a more flexible, gate-closed conformation in the nucleotide-bound state. These observations suggest that the increased ATP turnover for L18D homodimers may be due to disruption of stabilizing interactions between helix 1 and helix 5 of the ATP gate, allowing the gate to close more easily compared with WT, without prerequisite N-terminal β-strap release. Essentially, helix 1 destabilization eliminates one of the two high-barrier conformational changes within ATPase domain remodeling that are necessary for clamp cycling.

Our previous studies of WT:F6D/F8D heterodimers demonstrated that cross-subunit stabilization plays a role in driving Hsp90 ATPase activity (Fig. 7*C*) (15). In contrast, our ATPase assays for WT:L18D and WT:F6D/F8D/L18D heterodimers indicate that cross-subunit interactions do not stimulate or repress ATP hydrolysis beyond the expected synergy for WT Hsp90 dimers (5), as the heterodimer ATPase activity is an average between the WT homodimer and the respective L18D or F6D/F8D/L18D homodimer activities (Fig. 7, *D* and *E*). With this in mind, WT:L18D heterodimers have increased ATP turnover compared with WT homodimers because the enhanced activity of the L18D subunit increases the overall Hsp90 dimer ATP turnover (Fig. 7*D*). For the WT:F6D/F8D/L18D heterodimers, ATP turnover is half the activity of WT homodimers as the heterodimer contains only one functional WT subunit (Fig. 7*E*).

## Conclusions

Our findings provide a foundation to better understand both symmetric and asymmetric subunit control of the biological function of Hsp90 through manipulation of intrasubunit and intersubunit interactions and dynamics within the clamping cycle. We find that both symmetric and asymmetric subunit compositions can either stimulate or repress ATPase activity in homodimers and heterodimers (Fig. 5). However, the levels of stimulation and repression achieved by symmetric compositions (the diagonal values in the lower triangular matrix shown in Fig. 5) are larger in magnitude compared with what can be achieved by asymmetric subunit compositions (the off-diagonal values in the lower triangular matrix shown in Fig. 5). Furthermore, while ATPase activity is elevated for individual subunits within symmetric L18D homodimers, Aha1-mediated stimulation becomes impaired. In contrast, Aha1-mediated stimulation of asymmetric L18D:WT heterodimers remains similar to WT homodimers. Taken together, with previous work showing that cochaperone proteins, or post-translational modifications such as phosphorylation (31, 32, 33) or SUMOylation (34, 35) can stimulate individual subunits, our results indicate that both symmetric and asymmetric subunit composition for intact Hsp90 can be effective in modulating the conformational energy landscape of the chaperone clamping cycle. However, while asymmetric modification of the ATPase domain in intact Hsp90 can accelerate the chaperone clamping cycle, symmetric unmodified ATPase domains allow optimal cochaperone activation of Hsp90, whereas asymmetric modifications may interfere with cochaperone stimulation. The relevance of these findings for the interplay between Hsp90 post-translational modifications and interactions with cochaperones within a biological milieu remains an open question.

## Experimental procedures

### Expression and purification of Hsp90 and Aha1 proteins

Plasmids for Hsp90N-CYF3/L18D and Hsp90N-CYF3/F6D/F8D/L18D constructs were synthesized and inserted into the pHis-parallel vector by Genscript (*Saccharomyces cerevisiae*, Hsp82, UniProt KB: P02829). Coding sequences for intact Hsp90-CYF61, Hsp90-CYF110, Hsp90-E33A, Hsp90-F6D/F8D/CYF61, Hsp90-L18D/CYF61, and Hsp90-F6D/F8D/L18D/CYF61 were cloned into a pETd11His vector by Precision Biosciences. Isolated N-domain and intact Hsp90 constructs were expressed and purified as previously described (14, 36). Site-specific trifluoromethyl CYF (cysteine-trifluoroacetone derivative residue) probes were introduced by labeling cysteine mutants with trifluoroacetone (14, 37), and Aha1 cochaperone was expressed and purified as previously described (23).

### NMR spectroscopy

Preparation of protein samples for NMR, temperature-dependent CPMG experiments for Hsp90N, CPMG experiments for intact Hsp90-CYF110 and dynamic NMR experiments for intact Hsp90-L18D/CYF61, -F6D/F8D/L18D/CYF61, and -CYF110 were performed as previously described (14, 15, 36, 37). NMR experiments were conducted on a Bruker Avance III 700 (16.4 T) NMR spectrometer equipped with a TCI H&F/-CN cryogenic probe, and all ^19^F NMR spectra were processed using the Bruker TOPSPIN software. ^19^F dynamic NMR spectra for Hsp90-CYF110 upon AMP-PNP binding were acquired as previously described (14) and processed using the NMRPipe package (38). The resulting peak areas for the NM and clamp-closed states of Hsp90-CYF110 were fit to the rate laws for AMP-PNP binding, as previously described (14).

### Two-state fast exchange model for analyses of temperature-dependent CPMG relaxation dispersion experiments

CPMG ^19^F *R*_2_ relaxation dispersion curves were fit to a two-state fast exchange model using the expression (39, 40, 41, 42):

[1] $$R_{2.fast}\left(v_{CPMG}\right)=R_{2,0}+\frac{R_{ex}}{k_{ex}}\left[1-\left(\frac{{4v}_{CPMG}}{k_{ex}}\tanh\left[\frac{k_{ex}}{{4v}_{CPMG}}\right]\right)\right]$$

Where ν_CPMG_ is 1/(4τ_CPMG_) for a given CPMG element [τ_CPMG_ – 180° – τ_CPMG_ – τ_CPMG_ −180° – τ_CPMG_], τ_CPMG_ is the delay between inversion pulses, and *R*_2,0_ is the transverse relaxation rate given by the population weighted average for the *R*_2_ values of states *A* and *B*. In the fast exchange limit, *R*_ex_ is modeled exclusively as a single parameter; however, it is formally defined by:

[2] $$R_{ex}=p_{A}p_{B}{\Delta \omega }^{2}$$

where *p*_*A*_ and *p*_*B*_ are the steady-state populations and Δω is the chemical shift difference between states *A* and *B*. Temperature-dependent ^19^F *R*_2_ CPMG curves for Hsp90N-CYF3/L18D and Hsp90N-CYF3/F6D/F8D/L18D were fit using individual *R*_2,0_, *R*_ex_, and *k*_ex_ parameters. The fitted parameters were determined using global optimization of the sum of the squared differences between experimental and theoretical data points for each CPMG relaxation profile over 11 relaxation delays. Errors in the optimized parameters were determined using Monte Carlo parameter estimation.

### Hsp90 ATPase assays

ATP hydrolysis assays were performed as previously described (23) on 96-well plates with 100 μl reaction volumes using a BioTek Synergy 4 microplate reader with a path-length correction function. Assays were repeated four times with Hsp90 homodimers utilizing 5 μM of either Hsp90-CYF61, Hsp90-F6D/F8D/CYF61, Hsp90-L18D/CYF61, Hsp90-F6D/F8D/L18D/CYF61, or Hsp90-E33A. All Hsp90 heterodimer reactions maintained a total concentration of 10 μM Hsp90 using equimolar amounts of Hsp90-CYF61, Hsp90-F6D/F8D, Hsp90-L18D/CYF61, Hsp90-F6D/F8D/L18D/CYF61, or Hsp90-E33A to generate 1:2:1 combinations of dimeric Hsp90 consisting of homodimer1:heterodimer:homodimer2 subunit compositions, according to Pascal’s triangle. Homodimer1 and homodimer2 signify homodimers that are composed of only one of the two different subunits that make the heterodimer. Reactions involving Aha1 were performed in triplicate using a 3:1 ratio of Aha1:Hsp90 dimer with 6 μM Aha1 and 2 μM of Hsp90 dimer. Homodimer reactions with Aha1 utilized 2 μM of Hsp90-CYF61, Hsp90-L18D/CYF61, or Hsp90-F6D/F8D/L18D/CYF61. Heterodimer reactions with Aha1 utilized equimolar amounts of Hsp90-CYF61, Hsp90-L18D/CYF61, or Hsp90-F6D/F8D/L18D/CYF61 to generate 2 μM of 1:2:1 combinations of dimeric Hsp90 consisting of homodimer1:heterodimer:homodimer2 subunit compositions. All Hsp90 reactions were allowed to equilibrate for ∼1 h prior to addition to reaction plate wells. Reactions were then initiated by the addition of an ATP regeneration system (5 mM MgCl_2_, 1 mM DTT, 0.6 mM NADH, 2 mM ATP, 1 mM phosphoenol pyruvate, 2.5 μl of pyruvate kinase/lactate dehydrogenase [Sigma], and 4% dimethyl sulfoxide). To account for background ATPase activity, each assay condition was repeated while inhibiting Hsp90 with 150 μM NVP-AUY922 and subtracted from the mean of replicate wells containing dimethyl sulfoxide. The ATPase activity is expressed as a fraction of WT homodimer activity with error given as the standard deviation.

### Quantification of ATPase activity modulation by symmetric and asymmetric subunit composition

To separate the ATPase activities (expressed as fraction of WT activity) of the heterodimer and homodimer components from 1:2:1 homodimer:heterodimer:homodimer combinations that result from 1:1 stoichiometric mixing of two different Hsp90 subunits, the activity of the purely heterodimer component can be expressed as:

[3] $$hetD=\frac{\left(homhetmix\right)-\left(0.25\left(homD1\right)+0.25\left(homD2\right)\right)}{0.5}$$

where *hetD* is the calculated fractional WT ATPase activity due to heterodimer, *homD1* is the measured fractional ATPase activity for dimers of subunit1 from the heterodimer, *homD2* is the measured activity for dimers of subunit2 from the heterodimer, and *homhetmix* is the observed fractional WT ATPase activity for the combination homodimer1:heterodimer:homodimer2 with a stoichiometry of 1:2:1 derived from Pascal’s triangle. Subsequently, to determine the modulation of the fractional WT ATPase activity due to purely asymmetric heterodimers, we calculated the difference between the heterodimer contribution (*hetD*) and the ATPase activity measured for the 1:2:1 mixture (*homhetmix*):

[4] $$\Delta_{asymm}=hetD-homhetmix$$

Similarly, the symmetric modulation of the fractional WT ATPase activity for different Hsp90 homodimers can be expressed as:

[5] $$\Delta_{symm}=homD-WT$$

where *homD* is the fractional WT activity of a given homodimer, and *WT* is the normalized ATPase activity for WT homodimers.

### MD simulations for various Hsp90 ATPase domain mutants

MD simulations of apo and ATP-bound Hsp90N-CYF3, Hsp90N-CYF3/F6D/F8D, Hsp90N-CYF3/L18D, and Hsp90N-CYF3/F6D/F8D/L18D in explicit solvent were conducted using the AMBER suite of biomolecular simulation programs (36, 37, 43). Models for Hsp90N-CYF3 and Hsp90N-CYF3/F6D/F8D were created as previously described (15). Models for Hsp90N-CYF3/L18D and Hsp90N-CYF3/F6D/F8D/L18D were created by mutating L18 to aspartate within PyMol for the respective apo Hsp90N-CYF3 and Hsp90N-CYF3/F6D/F8D models (15). All models were solvated and relaxed using the OPC water model as previously described (15, 44) and production dynamics were run for 1 μs under constant pressure. Following MD simulations, we aligned the main chain N, Cα, and C atoms of residues 4 to 207 to calculate the dynamical cross-correlation matrices (45) for the Cα atoms at 10 ns steps over a 800 ns window. To determine the total correlation within specific protein regions, the values of the off-diagonal elements from the dynamical correlation matrix were summed while restricting the summation to avoid double counting the symmetric upper and lower matrices.

## Data availability

All data are contained within the article or supporting information. Source data are available upon request from the corresponding author L. S.

## Supporting information

This article contains supporting information (15).

## Conflict of interest

The authors declare that they have no conflicts of interest with the contents of this article.

## References

[1] Schopf F.H., Biebl M.M., Buchner J. (2017). The HSP90 chaperone machinery. Nat. Rev. Mol. Cell Biol..

[2] Pearl L.H. (2016). Review: the HSP90 molecular chaperone—an enigmatic ATPase. Biopolymers.

[3] Hartl F.U., Bracher A., Hayer-Hartl M. (2011). Molecular chaperones in protein folding and proteostasis. Nature.

[4] Biebl M.M., Buchner J. (2019). Structure, function, and regulation of the Hsp90 machinery. Cold Spring Harb. Perspect. Biol..

[5] Wegele H., Muschler P., Bunck M., Reinstein J., Buchner J. (2003). Dissection of the contribution of individual domains to the ATPase mechanism of Hsp90. J. Biol. Chem..

[6] Hessling M., Richter K., Buchner J. (2009). Dissection of the ATP-induced conformational cycle of the molecular chaperone Hsp90. Nat. Struct. Mol. Biol..

[7] Prodromou C. (2012). The ‘active life’ of Hsp90 complexes. Biochim. Biophys. Acta Mol. Cell Res..

[8] Krukenberg K.A., Street T.O., Lavery L.A., Agard D.A. (2011). Conformational dynamics of the molecular chaperone Hsp90. Q. Rev. Biophys..

[9] Li J., Sun L., Xu C., Yu F., Zhou H., Zhao Y. (2012). Structure insights into mechanisms of ATP hydrolysis and the activation of human heat-shock protein 90. Acta Biochim. Biophys. Sin. (Shanghai)..

[10] Ali M.M.U., Roe S.M., Vaughan C.K., Meyer P., Panaretou B., Piper P.W. (2006). Crystal structure of an Hsp90–nucleotide–p23/Sba1 closed chaperone complex. Nature.

[11] Richter K., Muschler P., Hainzl O., Buchner J. (2001). Coordinated ATP hydrolysis by the Hsp90 dimer. J. Biol. Chem..

[12] Schmid S., Götz M., Hugel T. (2016). Single-molecule analysis beyond dwell times: demonstration and assessment in and out of equilibrium. Biophys. J..

[13] Schulze A., Beliu G., Helmerich D.A., Schubert J., Pearl L.H., Prodromou C. (2016). Cooperation of local motions in the Hsp90 molecular chaperone ATPase mechanism. Nat. Chem. Biol..

[14] Lee B.L., Rashid S., Wajda B., Wolmarans A., LaPointe P., Spyracopoulos L. (2019). The Hsp90 chaperone: ^1^H and ^19^F dynamic nuclear magnetic resonance spectroscopy reveals a perfect enzyme. Biochemistry.

[15] Magnan B., Chen X.H., Rashid S., Minard A., Chau W., Uyesugi T. (2024). Asymmetric dynamics drive catalytic activation of the Hsp90 chaperone. J. Phys. Chem. B..

[16] Richter K., Reinstein J., Buchner J. (2002). N-terminal residues regulate the catalytic efficiency of the Hsp90 ATPase cycle. J. Biol. Chem..

[17] Retzlaff M., Hagn F., Mitschke L., Hessling M., Gugel F., Kessler H. (2010). Asymmetric activation of the Hsp90 dimer by its cochaperone Aha1. Mol. Cell..

[18] Cunningham C.N., Krukenberg K.A., Agard D.A. (2008). Intra- and intermonomer interactions are required to synergistically facilitate ATP hydrolysis in Hsp90. J. Biol. Chem..

[19] Prodromou C. (2000). The ATPase cycle of Hsp90 drives a molecular clamp’ via transient dimerization of the N-terminal domains. EMBO J..

[20] Amoah D.P., Hussein S.K., Johnson J.L., LaPointe P. (2025). Ordered ATP hydrolysis in the Hsp90 chaperone is regulated by Aha1 and a conserved post-translational modification. Protein Sci..

[21] Richter K., Moser S., Hagn F., Friedrich R., Hainzl O., Heller M. (2006). Intrinsic inhibition of the Hsp90 ATPase activity. J. Biol. Chem..

[22] Prodromou C., Roe S.M., Piper P.W., Pearl L.H. (1997). A molecular clamp in the crystal structure of the N-terminal domain of the yeast Hsp90 chaperone. Nat. Struct. Mol. Biol..

[23] Mercier R., Wolmarans A., Schubert J., Neuweiler H., Johnson J.L., LaPointe P. (2019). The conserved NxNNWHW motif in Aha-type co-chaperones modulates the kinetics of Hsp90 ATPase stimulation. Nat. Commun..

[24] Obermann W.M.J., Sondermann H., Russo A.A., Pavletich N.P., Hartl F.U. (1998). In vivo function of Hsp90 is dependent on ATP binding and ATP hydrolysis. J. Cell Biol..

[25] Panaretou B., Siligardi G., Meyer P., Maloney A., Sullivan J.K., Singh S. (2002). Activation of the ATPase activity of Hsp90 by the stress-regulated cochaperone Aha1. Mol. Cell..

[26] Liu Y., Sun M., Myasnikov A.G., Elnatan D., Delaeter N., Nguyenquang M. (2020). Cryo-EM structures reveal a multistep mechanism of Hsp90 activation by co-chaperone Aha1. bioRxiv.

[27] Meyer P. (2004). Structural basis for recruitment of the ATPase activator Aha1 to the Hsp90 chaperone machinery. EMBO J..

[28] Privalov P.L. (1979). Stability of proteins small globular proteins. Adv. Protein Chem..

[29] Makhatadze G.I., Privalov P.L. (1995). Energetics of protein structure. Adv. Protein Chem..

[30] Fersht A.R. (1993). Protein folding and stability: the pathway of folding of barnase. FEBS Lett..

[31] Mollapour M., Tsutsumi S., Donnelly A.C., Beebe K., Tokita M.J., Lee M.-J. (2010). Swe1Wee1-dependent tyrosine phosphorylation of Hsp90 regulates distinct facets of chaperone function. Mol. Cell..

[32] Mollapour M., Tsutsumi S., Truman A.W., Xu W., Vaughan C.K., Beebe K. (2011). Threonine 22 phosphorylation attenuates Hsp90 interaction with cochaperones and affects its chaperone activity. Mol. Cell.

[33] Xu W., Mollapour M., Prodromou C., Wang S., Scroggins B.T., Palchick Z. (2012). Dynamic tyrosine phosphorylation modulates cycling of the HSP90-P50CDC37-AHA1 chaperone machine. Mol. Cell.

[34] Mollapour M., Bourboulia D., Beebe K., Woodford M.R., Polier S., Hoang A. (2014). Asymmetric Hsp90 N domain SUMOylation recruits Aha1 and ATP-competitive inhibitors. Mol. Cell.

[35] Wolmarans A., Kwantes A., LaPointe P. (2019). A novel method for site-specific chemical SUMOylation: SUMOylation of Hsp90 modulates co-chaperone binding in vitro. Biol. Chem..

[36] Rashid S., Lee B.L., Wajda B., Spyracopoulos L. (2020). Nucleotide binding and active site gate dynamics for the Hsp90 chaperone ATPase domain from benchtop and high field ^19^F NMR spectroscopy. J. Phys. Chem. B..

[37] Rashid S., Lee B.L., Wajda B., Spyracopoulos L. (2019). Side-chain dynamics of the trifluoroacetone cysteine derivative characterized by ^19^F NMR relaxation and molecular dynamics simulations. J. Phys. Chem. B..

[38] Delaglio F., Grzesiek S., Vuister G.W., Zhu G., Pfeifer J., Bax A. (1995). NMRPipe: a multidimensional spectral processing system based on UNIX pipes. J. Biomol. NMR.

[39] Luz Z., Meiboom S. (1963). Nuclear magnetic resonance study of the protolysis of trimethylammonium ion in aqueous solution—order of the reaction with respect to solvent. J. Chem. Phys..

[40] Bloch F. (1946). Nuclear induction. Phys. Rev..

[41] McConnell H.M. (1958). Reaction rates by nuclear magnetic resonance. J. Chem. Phys..

[42] Binsch G. (1968). The study of intramolecular rate processes by dynamic nuclear magnetic resonance. Top. Stereochem..

[43] Cornell W.D., Cieplak P., Bayly C.I., Gould I.R., Merz K.M., Ferguson D.M. (1995). A second generation force field for the simulation of proteins, nucleic acids, and organic molecules. J. Am. Chem. Soc..

[44] Izadi S., Anandakrishnan R., Onufriev A.V. (2014). Building water models: a different approach. J. Phys. Chem. Lett..

[45] Hünenberger P.H., Mark A.E., Van Gunsteren W.F. (1995). Fluctuation and cross-correlation analysis of protein motions observed in nanosecond molecular dynamics simulations. J. Mol. Biol..

