# Supplementary material for: Symmetric stimulation of Hsp90 catalyzed ATP hydrolysis through enhanced active site gate dynamics
Source: J Biol Chem. 2025 May 21;301(6):110262. doi: 10.1016/j.jbc.2025.110262 (PMC12205646; doi:10.1016/j.jbc.2025.110262)
Supplement: Supplementary Information [file mmc1.pdf]

Symmetric stimulation of Hsp90 catalyzed ATP hydrolysis through enhanced active site gate dynamics

Breanna Magnan[†]*, Thomas Dumont*[†]*, Suad Rashid*[†]*, Paul LaPointe*[‡] *and Leo Spyracopoulos*[†,*].

[†] Department of Biochemistry, University of Alberta, Edmonton, AB, T6G 2H7, Canada

[‡] Department of Cell Biology, University of Alberta, Edmonton, AB, T6G 2H7, Canada

*Corresponding author: leo.spyracopoulos@ualberta.ca

Figure S1 – Structural models of apo Hsp90N and AMP-PNP bound intact Hsp90 with ^{19}F NMR spectra for apo Hsp90N-CYF3, Hsp90N-CYF3/L18D, Hsp90N-CYF3/F6D/F8D, Hsp90N-CYF3/F6D/F8D/G123P, and Hsp90N-CYF3/F6D/F8D/L18D at 25 °C

Figure S2 – Snapshots and dynamic correlation matrices for MD simulations of Hsp90N-CYF3 and Hsp90N-CYF3/L18D in the absence and presence of ATP.

Figure S3 – ^{19}F NMR spectrum for apo Hsp90N-CYF3/F6D/F8D/L18D at 10 °C and corresponding atomic models for various ATPase domain conformations.

Figure S4 – Snapshots and dynamic correlation matrices for MD simulations of Hsp90N-CYF3/F6D/F8D, and Hsp90N-CYF3/F6D/F8D/L18D in the absence and presence of ATP.

Figure S5 – Structural model of AMP-PNP bound intact Hsp90 with ^{19}F NMR data for Hsp90-CYF61 and Hsp90-CYF110 in the absence and presence of ATP.

Table S1 – Fitted parameters for two-site exchange in Hsp90N-CYF3/L18D and Hsp90N-CYF3/F6D/F8D/L18D.

Table S2 – ATPase activity for L18D mutant homo- and heterodimers in the absence and presence of Aha1.

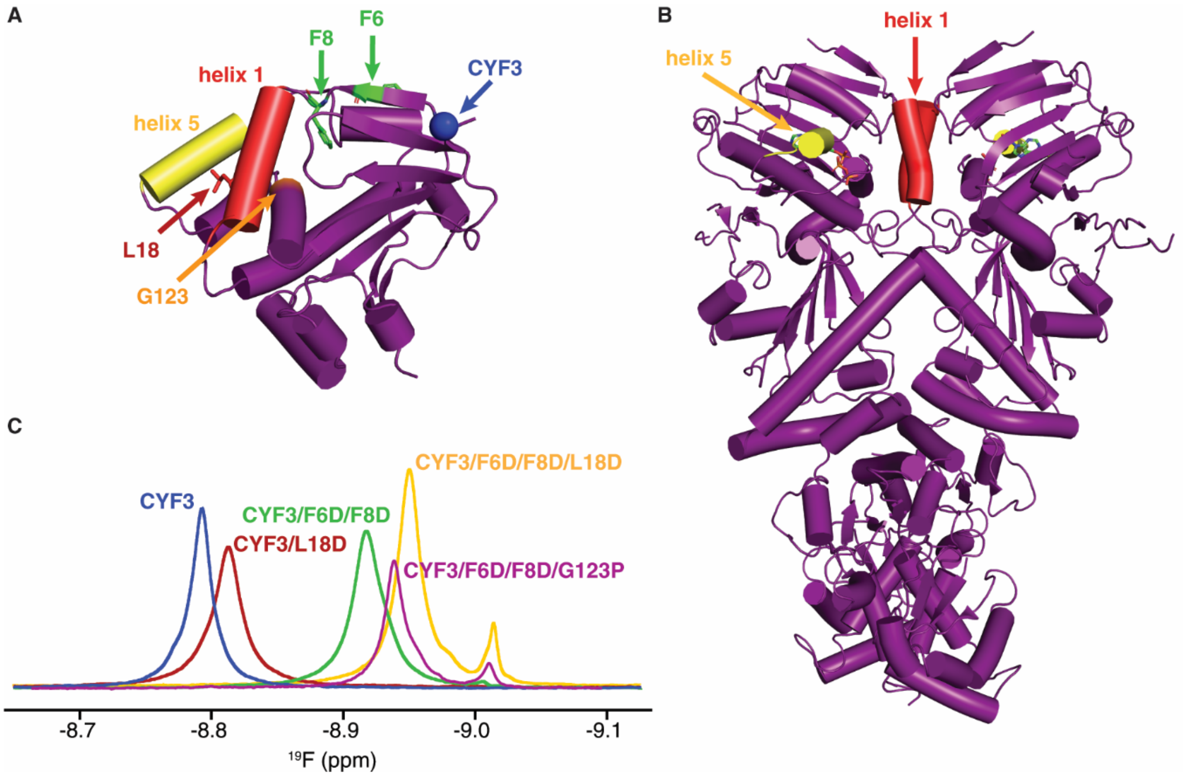

Figure S1. ^{19}F chemical shift changes resulting from N-terminal β-strap, α-helix 1, and ATP gate mutations. Structure of apo Hsp90N (A; 1AH6) with helix 1 shown in red and helix 5 shown in yellow. The Cα atom of CYF3 is shown as a sphere and the F6, F8, L18, and G123 residues are shown in the stick representation. (B) Structure of AMP-PNP bound intact Hsp90 (2CG9) with helix 1 shown in red and helix 5 shown in yellow. AMP-PNP is shown in the ball and stick representation. (C) ^{19}F NMR spectra for Hsp90N-CYF3 (blue), Hsp90N-CYF3/L18D (red), Hsp90N-CYF3/F6D/F8D (green), Hsp90N-CYF3/F6D/F8D/G123P (purple), and Hsp90N-CYF3/F6D/F8D/L18D (yellow) at 25°C in the absence of nucleotide. Spectra of apo Hsp90N-CYF3, Hsp90N-CYF3/F6D/F8D, and Hsp90N-CYF3/F6D/F8D/G123P were previously acquired (15).

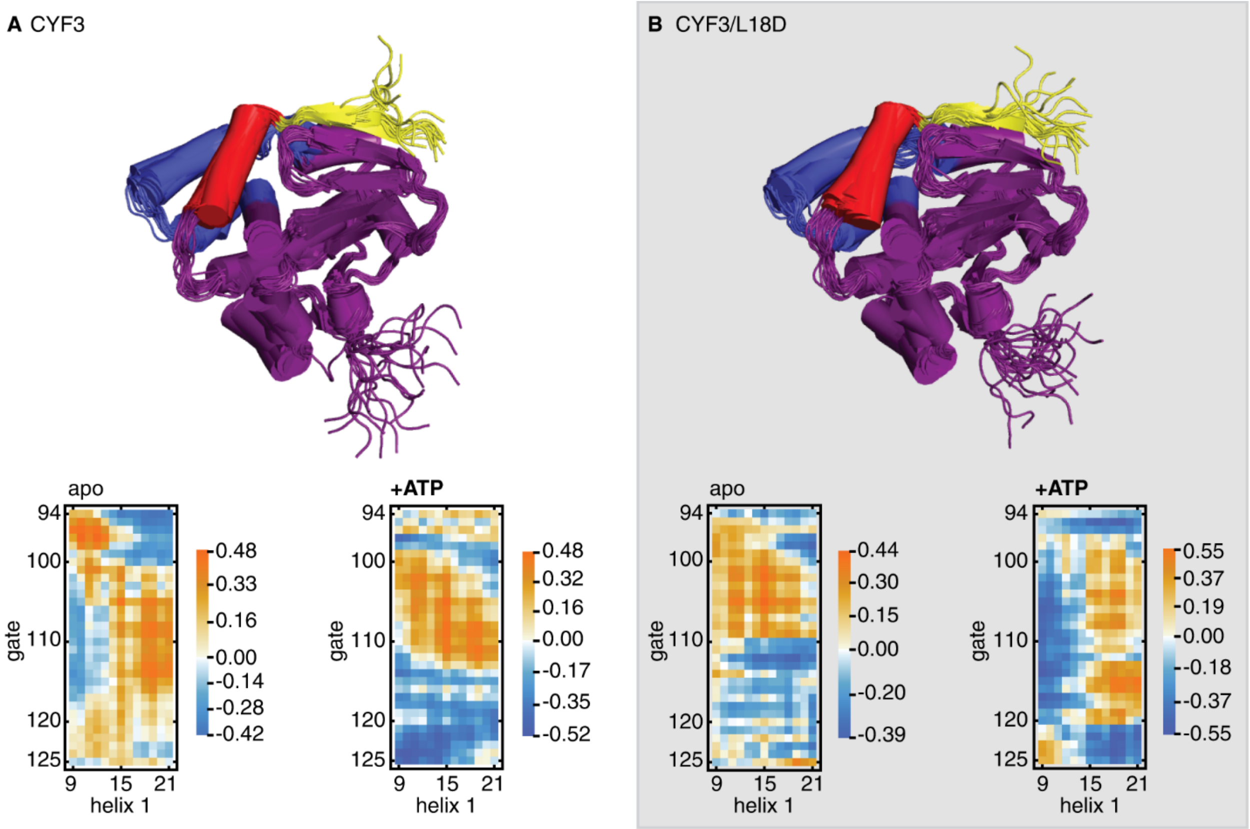

Figure S2. MD simulations reveal coupling between helix 1 and the ATP gate. Representative snapshots from 1 μs MD simulations for Hsp90N-CYF3 (A) and Hsp90N-CYF3/L18D (B). The N-terminal strap is shown in yellow, helix 1 is shown in red, and gate regions are shown in blue. Dynamical cross-correlation maps for pairs of Cα atoms from helix 1 and the ATP gate for Hsp90N-CYF3 (A) and Hsp90N-CYF3/L18D (B) in the absence (bottom left panel) and presence of ATP (bottom right panel). Uncorrelated motions for Cα atoms are shown in white, positively correlated motions are shown in orange, and negatively correlated motions are shown in blue.

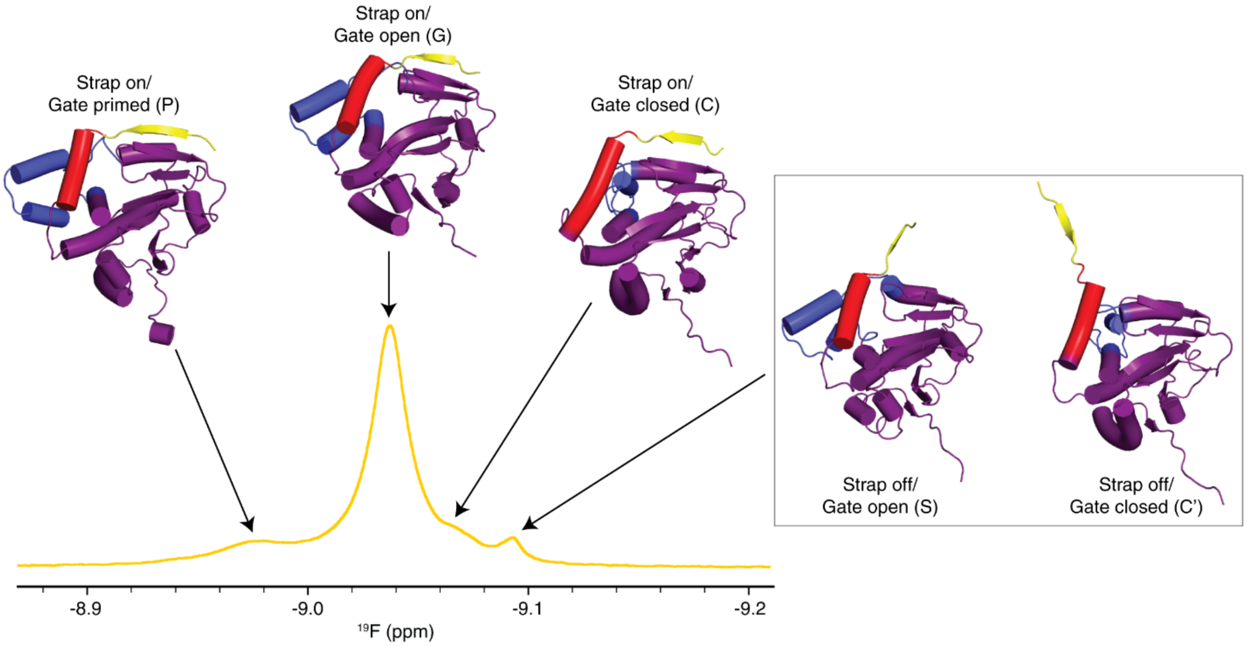

Figure S3. ^{19}F NMR reveals multiple ATPase domain conformations. ^{19}F NMR spectrum for Hsp90N-CYF3/F6D/F8D/L18D at 10 °C in the absence of nucleotide, with resonance peaks labeled according to atomic models for the gate primed (P), ground (G), strap-on/gate-closed (C), strap-off/gate-open (S), and strap-off/gate-closed (C') states. The N-terminal strap is shown in yellow, helix 1 is shown in red, and gate regions are shown in blue.

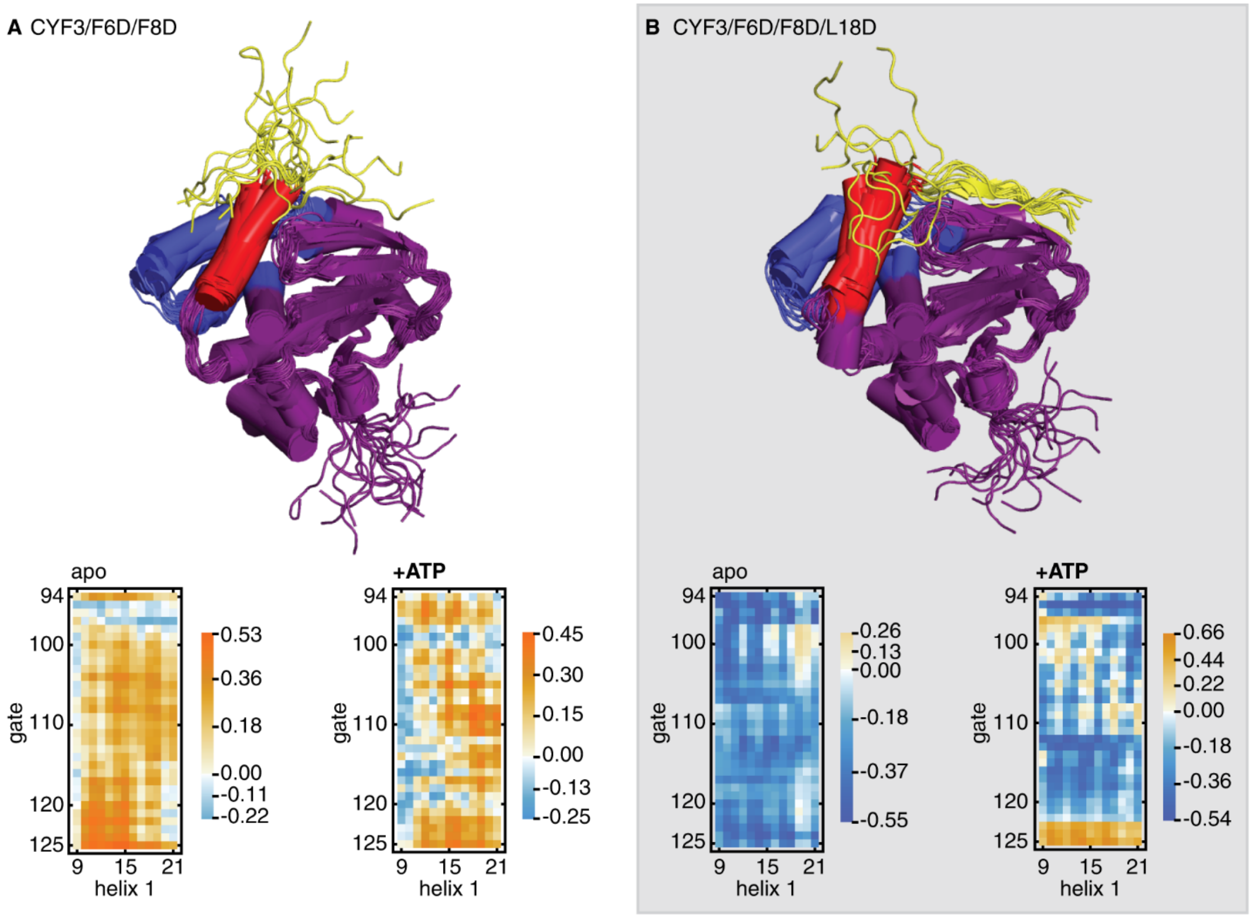

Figure S4. MD simulations reveal coupling between helix 1 and the β-strap. Representative snapshots from 1 μs MD simulations for Hsp90N-CYF3/F6D/F8D (A) and Hsp90N-CYF3/F6D/F8D/L18D (B) in the absence of nucleotide (top panel). The N-terminal strap is shown in yellow, helix 1 is shown in red, and gate regions are shown in blue. Dynamical cross-correlation maps for pairs of Cα atoms from helix 1 and the ATP gate for Hsp90N-CYF3/F6D/F8D (A) and Hsp90N-CYF3/F6D/F8D/L18D (B) in the absence (bottom left panel) and presence of ATP (bottom right panel). Uncorrelated motions for Cα atoms are shown in white, positively correlated motions are shown in orange, and negatively correlated motions are shown in blue.

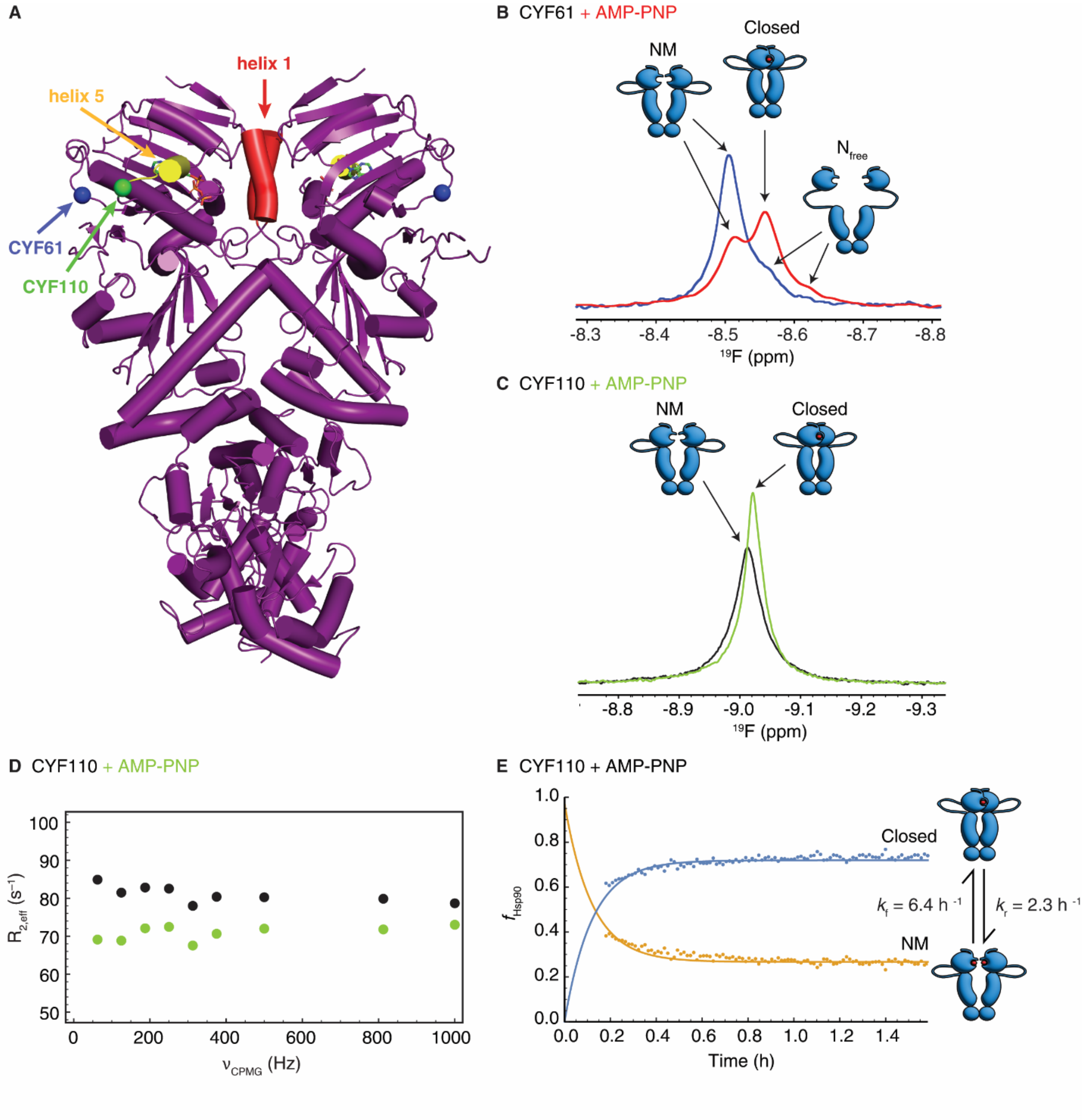

Figure S5. Different conformational states of intact Hsp90. (A) Structure of AMP-PNP bound intact Hsp90 (2CG9) with helix 1 shown in red and helix 5 shown in yellow. AMP-PNP is shown in the ball and stick representation with the Cα atoms of the CYF61 and CYF110 ^{19}F probes shown as blue and green spheres, respectively. ^{19}F NMR spectra of Hsp90-CYF61 (B) and CYF110 (C) in the absence and presence of AMP-PNP, with resonance peaks labeled according to models for the NM-associated, free N-domain, and clamp-closed conformational states. (D)

^{19}F NMR CPMG relaxation dispersion profiles at 657 MHz and 25 °C for Hsp90-CYF110 in the absence (black) and presence (green) of AMP-PNP. (E) Hsp90-CYF110 ^{19}F NMR peak areas for the clamp closed and NM-associated states were fit to the integrated rate laws for the Hsp90 cycle for the NM (orange) and closed (blue) states. Spectra of apo Hsp90-CYF61 and AMP-PNP bound Hsp90-CYF61 was previously acquired (15).

Temperature (°C)	N-CYF3/L18D	N-CYF3/F6D/F8D/L18D
	k_{ex} (s^{-1})	k_{ex} (s^{-1})
10	333 ± 22	86 ± 54
15	697 ± 44	213 ± 25
20	570 ± 42	234 ± 16
25	545 ± 54	406 ± 21
30	603 ± 74	855 ± 101

Table S1. Fitted parameters for two-site exchange in Hsp90N-CYF3/L18D and Hsp90N-CYF3/F6D/F8D/L18D. Exchange rates (k_{ex}) determined from fits of CPMG relaxation dispersion data for Hsp90N-CYF3/L18D and Hsp90N-CYF3/F6D/F8D/L18D to a two-state kinetic mode. The errors for the fitted parameters were estimated using Monte Carlo analyses.

	Fraction wildtype ATPase activity	
	– Aha1	+ Aha1
WT	1.00 ± 0.04	20.0 ± 0.2
L18D	2.17 ± 0.02	12.6 ± 0.8
F6D/F8D/L18D	0.08 ± 0.05	0.05 ± 0.09
WT:L18D	1.62 ± 0.03	19 ± 1
WT:F6D/F8D/L18D	0.5 ± 0.1	7.23 ± 0.1
L18D:F6D/F8D/L18D	0.72 ± 0.09	4.2 ± 0.4

Table S2. ATPase activity for L18D mutant homo- and heterodimers in the absence and presence of Aha1. ATPase activity is shown as a fraction of wildtype homodimer activity. The errors indicate the standard deviation for experiments conducted in triplicate.
